# Reversible Bilateral Basal Ganglia Lesions Due to Multifactorial Toxic-Metabolic Disorders

**DOI:** 10.7759/cureus.38733

**Published:** 2023-05-08

**Authors:** Octávia Costa, Joana Pinto, Ana Filipa Santos

**Affiliations:** 1 Neurology, Hospital de Braga, Braga, PRT

**Keywords:** acute confusional state, toxic exposures, diabetes mellitus type 2, hyperintense basal ganglia, basal ganglia disease

## Abstract

Bilateral basal ganglia lesions can include a wide variety of etiologies, including metabolic, toxic, degenerative, vascular, inflammatory, infectious, and neoplastic etiology.

We present a case of a 78-year-old man who was hospitalized with acute behavioral changes and psychomotor slowing. His medical history included diabetes mellitus, arterial hypertension, and prostate adenocarcinoma. In his spare time, he was a pigeon fancier and regularly burned waste (including diapers) outside his home.

In the initial evaluation, he was hypertensive, drowsy, disoriented in time and space, dysarthric, and with global bradykinesia.

From the research carried out, we stand out the following: brain magnetic resonance imaging showing bilateral hyperintensity of the basal ganglia on T2/fluid-attenuated inversion recovery, with foci of hypersignal on T1 without diffusion restriction or contrast enhancement; CSF presenting 15 cells/uL, without other alterations; analytical results presenting hypernatremia (171 mEq/L), creatinine at 3.5 mg/dL, hyperglycemia (always <300 mg/dL), and slightly elevated C-reactive protein and anticardiolipin antibodies in addition to thrombocytopenia (107,000).

After correcting the metabolic disturbances and evading the identified toxic substances, magnetic resonance imaging showed regression of the lesions, and the patient returned to a normal state.

The functions of the basal ganglia are complex, requiring increased use of glucose and oxygen, therefore presenting a high metabolic activity, which makes them vulnerable to various metabolic changes. We report a rare case affected by symmetrical lesions in the basal ganglia and presenting an acute onset of altered mental status with behavioral alterations, related to hyperglycemia, acute kidney injury, hypertension, and exposure to toxic substances (smoke from bonfires and/or toxic chemical components). Complete clinical recovery, remaining negative investigation, and regression of the lesions support our diagnosis.

## Introduction

Bilateral basal ganglia lesion is a rare syndrome that can occur in multiple diseases, including systemic metabolic abnormalities, toxic poisoning, neoplasms, and degenerative, vascular, inflammatory, and infectious conditions. This case aims to discuss the relationship between the toxic-metabolic disorders of our patient, the signs and symptoms, and changes in imaging. Although metabolic and vascular factors are considered important in the pathophysiology, it remains unclear [1].

## Case presentation

An independent 78-year-old man was admitted to the neurology department for acute confusional syndrome, occurring on the morning of the admission day, and psychomotor lentification. According to family members, he drove his car over a roundabout and presented repetitive speech and temporal disorientation.

His medical background included diabetes mellitus, arterial hypertension, and prostate adenocarcinoma. In his free time, he was a pigeon fancier and regularly burned waste (including diapers) outside the house, exposing himself to smoke. He had no history of alcohol abuse. No new medication had been started. There was no recent travel or animal bite history. No similar occurrences in the past were recorded.

On admission examination, he was hypertensive (systolic tension = 190 mmHg) and drowsy, with difficult maintenance of attention, spatial and temporal disorientation, moderate dysarthria, global bradykinesia, and increased limb tone. He presented no involuntary movements, no focal neurological signs, or fever.

Blood analysis showed hypernatremia (maximum of 171 mEq/L), acute kidney disease (maximum creatinine of 3.5 mg/dL), and hyperglycemia (always <300 mg/dL) that were properly corrected. He also presented an elevation of C-reactive protein (20.9 mg/L) and thrombocytopenia (107,000/uL). Immunological studies that included antinuclear antibodies, anti-extractable nuclear antigen antibodies, antithyroid peroxidase, antithyroglobulin, and anti-transglutaminase were normal, except for a slight rise of anti-cardiolipin and beta-2 glycoprotein antibodies (only one measurement).

No antineuronal antibodies were identified either in cerebrospinal fluid (anti-NMDAR, AMPAR, GABAbR, GABAaR, DPPX, LGI1, CASPR2, mGluR, and GAD65 antibodies) or serum (Yo/PCA1, Hu/ANNA1, Ri/ANNA2, Tr/DNER, zic4, titin, SOX1, recoverin, PNMA2/Ma2/Ta, CV2/CRMP5, amphiphysin, and GAD65 antibodies). Blood gas and urinary analysis were normal.

**Figure 1:**
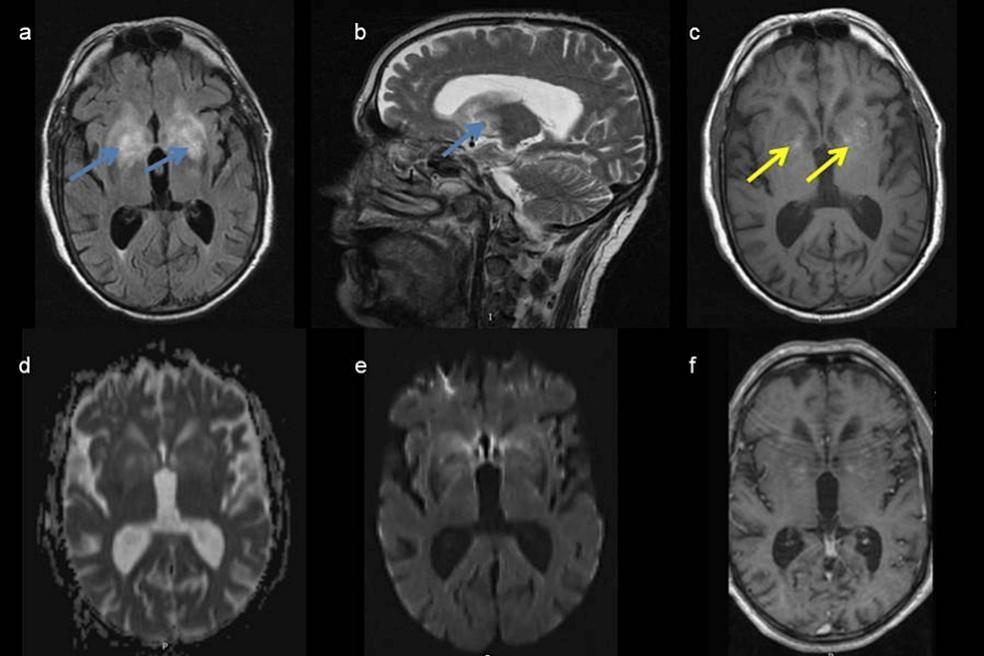
Brain magnetic resonance imaging in the acute phase showing bilateral lenticulocapsular and caudate nuclei hyperintensity (blue arrow) on T2/fluid-attenuated inversion recovery (a, b), hyperintensity foci on T1 sequence (c, yellow arrow) without restriction to diffusion (d) or contrast enhancement (e, f).

Brain magnetic resonance imaging showed bilateral lenticulocapsular and caudate nuclei hyperintensity on T2/fluid-attenuated inversion recovery (FLAIR), with T1 hypersignal foci without restriction to diffusion or contrast enhancement. Magnetic resonance imaging angiography was normal (Figure 1).

Cerebrospinal fluid presented mild pleocytosis (15 cells/uL) with normal or negative proteins, glucose, polymerase chain reaction for viruses, and mycobacteriological and microbacteriological cultures. Electroencephalogram monitoring showed no alterations.

A workup for an occult neoplasm that included thoracoabdominopelvic computed tomography, positron emission tomography, thyroid and testicular ultrasound, and tumoral markers was unremarkable.

Facing the remote possibility of autoimmune encephalitis, even before the analytical study was ready, the patient was treated with intravenous human immunoglobulins, with no improvement. Steroids were not started, taking into consideration the possibility of a central variant posterior reversible encephalopathy syndrome.

**Figure 2:**
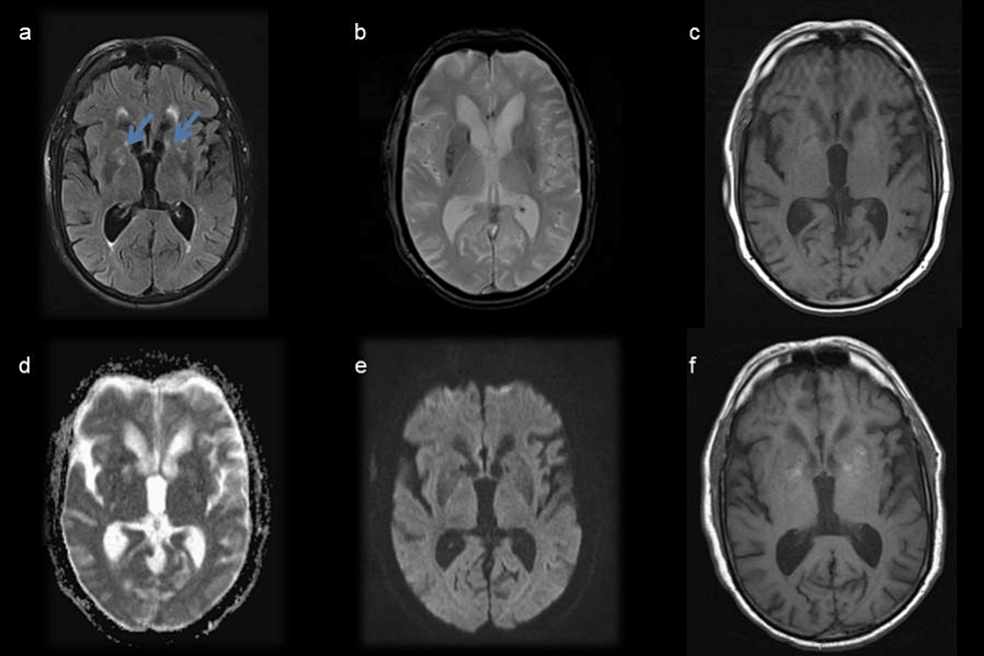
Brain magnetic resonance imaging at six weeks after initial symptoms showing attenuation of bilateral symmetrical heterogeneous lenticulocapsular and caudate nuclei hyperintensity (blue arrow) on T2/fluid-attenuated inversion recovery (a), without hyperintensity foci on T1 sequence (c), microhemorrhages (b), restriction to diffusion (d, e), or contrast enhancement (f).

After correcting the above-mentioned metabolic disorders and evading the previously identified toxics, magnetic resonance imaging showed lesions in the same location but more heterogeneous with areas suggesting resolution of lesions (Figure 2). The patient returned to his usual state.

## Discussion

We report a rare case affected with symmetrical basal ganglia lesions and exhibiting acute onset of altered mental status and behavioral changes, related to hyperglycemia, acute kidney injury, hypertension, and exposure to toxic substances such as smoke and other chemical components. Lesions and clinical symptoms disappeared with the correction of metabolic disorders and removal of toxic exposure, similar to those reported in other patients with acute bilateral basal ganglia lesions [2]. The full clinical recovery, remaining negative work-up, and lesions regression support our diagnosis.

Some of the toxic and metabolic disorders that affect the central nervous system present a typical pattern involvement that can suggest the etiology. These patterns include symmetrical basal ganglia and/or thalami, cortical gray matter, periventricular white matter, corticospinal tract, corpus callosum, parieto-occipital vasogenic edema, and central pons involvement [1]. Asymmetric, focal, or discrete lesions affecting only part of the basal ganglia tend to indicate involvement by infections or neoplasms.

Some of the suggestive causes of bilateral basal ganglia lesions include carbon monoxide intoxication, hypoxia, toxin exposition, metabolic disorders, vasculitis, and encephalitis [3]. Oftentimes, the diagnosis is not straightforward, and the correlation of typical imaging features with clinical and laboratory data can help make the correct diagnosis.

In our case, the partial reversibility of the lesion was possible. As previously reported, the reversibility of these lesions is related to the intensity and duration of exposure [2-4].

Although it is a rare syndrome, we intend to highlight that metabolic disorders and exposure to certain toxins can cause damage to the central nervous system, in particular, the basal ganglia. Their high metabolic activity and increased glucose and oxygen utilization make them vulnerable to metabolic abnormalities and many systemic or generalized disease processes. We alert that this recognition is essential as treating and controlling these disorders allows reversibility of the injuries.

## Conclusions

Metabolic disorders and exposure to certain toxins can cause damage to the central nervous system, in particular, the basal ganglia. Their high metabolic activity and increased utilization of glucose and oxygen make them vulnerable to metabolic abnormalities and many systemic or generalized disease processes. We alert that this recognition is essential as the treatment and control of these disorders allow reversibility of the injuries.
